# Complete mitochondrial genome of the Andean morphotype of *Anastrepha fraterculus* (Wiedemann) (Diptera: Tephritidae)

**DOI:** 10.1080/23802359.2017.1307706

**Published:** 2017-04-07

**Authors:** Juan P. Isaza, Juan F. Alzate, Nelson A. Canal

**Affiliations:** a Grupo de parasitología, Facultad de Medicina, Universidad de Antioquia, Medellín, Colombia, Centro Nacional de Secuenciación Genómica – CNSG, Sede de Investigación Universitaria SIU, Universidad de Antioquia, Medellín, Colombia;; b Universidad del Tolima, Facultad de Ingenieria Agronómica, Barrio Santa Helena Parte Alta, Ibagué, Tolima, Colombia

**Keywords:** Genomic, South American fruit fly, phylogeny

## Abstract

The South America fruit fly *Anastrepha fraterculus s.l.* is an important pest of fruits in Latin America and it is really a complex with at least eight cryptic species. In this work, we report the complete mitochondrial genome for the Andean morphotype of *A. fraterculus*. The mitochondrial genome is 16,739 nucleotides in size; includes 13 protein-coding genes, 22 tRNA genes, and 2 rRNA genes. Phylogenetic analysis was performed using all the protein-coding genes with other 19 species from Tephritidae.

*Anastrepha fraterculus* is one of the most economically important species of fruit flies in the tropical region of America, mainly because the wide variety of plants that it attacks and its quarentenarian restrictions (Canal et al. 2015). *Anastrepha fraterculus* is a nominal species that represents a complex with at least eight cryptic species with morphological, genetical, chemical, and biological differences (Hernández-Ortiz et al. 2012, 2015; Vaníčková et al. 2015). The Andean morphotype is located in the Andes mountain from the south of Colombia to Venezuela, at altitudes ranging from 1500 to 2500 m (Castañeda et al. 2010; Hernández-Ortiz et al. 2012). The mitochondrial genome provides valuable information to better understand the phylogenetic relationship between the species of *A. fraterculus* complex and within genus. In this study, we report the mitochondrial genome of the Andean morphotype of *A. fraterculus* and it is the first mitogenome for the genus *Anastrepha*.

Specimens of *A. fraterculus* were obtained from infested coffee berries in Ibagué, Tolima, Colombia (04° 24′ 53,5ÊN 75° 18′ 50,6ÊW); the body of the specimens analyzed was totally processed, but specimens voucher collected in the same place and data are deposited in the Museo del Laboratorio de Entomologia de la Universidad del Tolima (MEN – UT), and specimens from the same place had been used for other studies (Hernández-Ortiz et al. 2012; Canal et al. 2015; Hernández-Ortiz et al. 2015; Vaníčková et al. 2015). The genomic DNA was extracted from the thorax of the flies and a Whole Genome Shotgun (WGS) sequencing strategy in the platform 454 FLX + was implemented (Roche, Basel, Switzerland) to sequence the mitochondrial genome. The genome was *de novo* assembled using NEWBLER v2.9 (Margulies et al. 2005), and contigs carrying mitochondrial-coding sequences were extracted from the whole genome contig dataset using BLASTN (Altschul et al. 1990) against the NCBI nucleotide (NT) database. One contig of 16,739 nucleotides was identified as the mitochondrial genome. It was automatically annotated via MITOS webserver (mitos.bioinf.uni-leipzig.de/index.py) and manually curated in ARTEMIS v16 (Rutherford et al. 2000). Conflictive genome regions were confirmed through PCR and capillary sequencing using an ABI 3730XL. The complete mitochondrial genome is 16,739 nucleotides and is available in the GeneBank database with the accession number KX926433. It contains a low GC content (22.9%) and the typical metazoan 13 protein-coding genes (PCGs), 22 transfer RNAs, and 2 ribosomal RNAs. According to the data available in the GenBank, a comparative genomic analysis between *A. fraterculus* and species from the genera *Bactrocera*, *Ceratitis*, and *Procecidochares* showed that the sinteny in the mitochondrial genome was conserved through the Tephritidae family. In addition, the phylogenetic position of *A. fraterculus* inside Tephritidae family was established (Figure 1) and it is located as a sister clade of *Bactrocera* spp.

**Figure 1.**
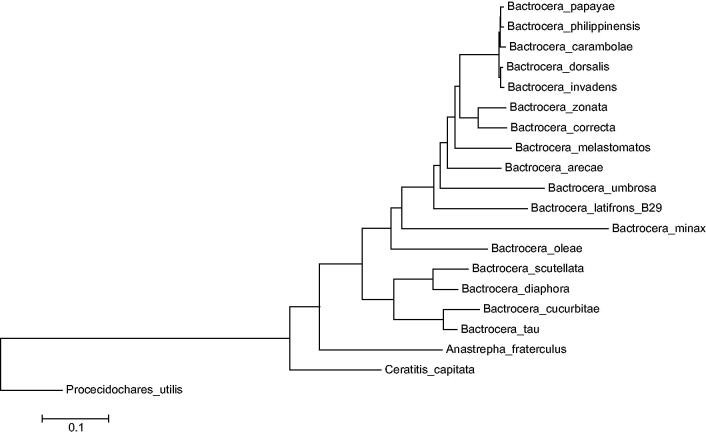
Phylogenetic position of *A. fraterculus*. Maximum-likelihood tree inferred from the nucleotide sequence of 13 PCGs in the mitogenome. Tree topology is based on the General Time Reversible substitution model with rates among sites Gamma distributed with invariants sites. Numbers represents bootstrap score from 1000 replicates. Phylogenetic analyses were conducted in MEGA v6 (Tamura et al. 2013). GenBank accession numbers are as follows: *A. fraterculus* (KX926433), *C. capitata* (NC_000857), *Bactrocera arecae* (NC_028327), *B. carambolae* (NC_009772), *B. correcta* (NC_018787), *B. cucurbitae* (NC_016056), *B. diaphora* (NC_028347), *B. dorsalis* (NC_008748), *B. invadens* (NC_031388), *B. latifrons* (NC_029466), *B. melastomatos* (NC_029467), *B. minax* (NC_014402), *B. oleae* (NC_005333), *B. papaya* (NC_009770), *B. philippinensis* (NC_009771), *B. scutellata* (NC_027254), *B. tau* (NC_027290), *B. umbrosa* (NC_029468), *B. zonata* (NC_027725), and *Procecidochares utilis* (NC_020463).
